# Exploring Pragmatic Deficits in Relation to Theory of Mind and Executive Functions: Evidence from Individuals with Right Hemisphere Stroke

**DOI:** 10.3390/brainsci13101385

**Published:** 2023-09-29

**Authors:** Dimitrios Tsolakopoulos, Dimitrios Kasselimis, Nikolaos Laskaris, Georgia Angelopoulou, Georgios Papageorgiou, Georgios Velonakis, Maria Varkanitsa, Argyro Tountopoulou, Sofia Vassilopoulou, Dionysis Goutsos, Constantin Potagas

**Affiliations:** 1 Neuropsychology and Language Disorders Unit, 1st Department of Neurology, Eginition Hospital, National and Kapodistrian University of Athens, 72-74 Vas. Sofias Av., 11528 Athens, Greece; 2 Department of Psychology, Panteion University of Social and Political Sciences, 17671 Athens, Greece; 3 Department of Industrial Design and Production Engineering, School of Engineering, University of West Attica, 12243 Athens, Greece; 4 Second Department of Radiology, Attikon General University Hospital, National and Kapodistrian University of Athens, 15772 Athens, Greece; 5 Center for Brain Recovery, Sargent College of Health and Rehabilitation Sciences, Boston University, Boston, MA 02215, USA; 6 Stroke Unit, 1st Department of Neurology, Eginition Hospital, National and Kapodistrian University of Athens, 15772 Athens, Greece; 7 Department of Linguistics, National and Kapodistrian University of Athens, 15772 Athens, Greece

**Keywords:** pragmatics, Theory of Mind, executive functions, right hemisphere stroke, communication disorders

## Abstract

Research investigating pragmatic deficits in individuals with right hemisphere damage focuses on identifying the potential mechanisms responsible for the nature of these impairments. Nonetheless, the presumed shared cognitive mechanisms that could account for these deficits have not yet been established through data-based evidence from lesion studies. This study aimed to examine the co-occurrence of pragmatic language deficits, Theory of Mind impairments, and executive functions while also exploring their associations with brain lesion sites. Twenty-five patients suffering from unilateral right hemisphere stroke and thirty-seven healthy participants were recruited for this study. The two groups were tested in pragmatics, Theory of Mind, and executive function tasks. Structural imaging data were also obtained for the identification of the lesion sites. The findings of this study suggest a potential convergence among the three aforementioned cognitive mechanisms. Moreover, we postulate a hypothesis for a neural circuitry for communication impairments observed in individuals with right hemisphere damage.

## 1. Introduction

Although the left hemisphere is undoubtedly associated with the primary language components, such as semantic and phonological processing, there is plenty of evidence indicating the role of the right hemisphere in the understanding and production of words and sentences at a different cognitive level, which is referred to as pragmatics. Pragmatics is the use of language to convey communicative meaning in a given context [1,2,3,4,5].

Older lesion studies on the subject have linked right hemisphere lesions with deficits in processing of figurative speech, i.e., patients tend to choose the literal interpretation of metaphorical sentences, while neurotypical participants perceive them as non-literal/metaphorical [6]. Although it seems that the right hemisphere is not uniquely involved in the process of metaphorical language, right hemisphere lesions have been shown to unequivocally affect this ability [7]. Right brain damage (RBD) has also been linked to difficulties in processing humor, particularly in the form of inability to understand the coherence of a joke [8,9] and understanding the connection between words based on the secondary and metaphorical meaning [10]. Furthermore, RBD patients appear to have deficits regarding in-context comprehension, showing difficulty in the process of the macrostructure of a text, as they exhibit more confabulations and distort aspects of the story. Difficulties in global coherence of a narrative, production of inefficient narratives in terms of understanding the central theme and conclusion, as well as impaired procedural discourse in picture description and everyday conversation are also exhibited [11,12,13,14]. Finally, difficulties in distinguishing lies from jokes [15], as well as difficulties in processing of sarcasm in various language structures [16,17], have been reported. These communication deficits seem to affect the everyday functionality of the patients [18].

Despite the available information on the relationship between deficits in pragmatic skills and right hemisphere lesions, a firm conclusion on the subject has yet to be drawn. Not all RBD patients show the same communication deficits, nor do they have the same deficit severity. It is estimated that 50–80% of RBD individuals will suffer from communication deficits, among which there is a subgroup of patients exhibiting impaired processing of pragmatics [19,20]. Overall, the ambiguous relationship between impaired pragmatic skills and brain damage affecting the right hemisphere could be attributed to two main factors. First, the intrinsic heterogeneity of the pragmatic aspect of language, especially when observed and measured in RBD patients of various pathologies [21]. The pattern of pragmatic deficits seems to differ among disorders [22]. To deal with this issue, scholars have coined the term “apragmatism” as an umbrella term which may include the various deficits within the pragmatic spectrum [23]. The second factor refers to the underlying pathological cognitive mechanisms related to pragmatic deficits, i.e., Theory of Mind and executive functions [24,25,26,27,28].

Regarding the first factor (i.e., heterogeneity of pragmatics), although pragmatics consists of various linguistic components, many neuropsychological studies investigating communication deficits in RBD patients usually treat pragmatics as a unitary cognitive process by evaluating a single pragmatic aspect. This may result in insufficient conclusions regarding the communicational profiles of RBD patients [29], since patients may exhibit pragmatic deficits beyond the subcomponent being investigated [20]. This issue becomes more apparent when RBD patients are evaluated with a variety of communication tests, examining different aspects of pragmatics, where they appear to split into contrasting groups on the basis of the different deficits demonstrated [20,30]. These studies reveal distinct profiles of communication deficits, with some RBD patients demonstrating deficits in some subcomponents of pragmatics and paralinguistic language structures, others exhibiting deficits across the spectrum of pragmatic language, and patients who do not exhibit any communication deficits at all [20]. Overall, even though research on this topic has established that RBD individuals vary in terms of their communication abilities, and at least a portion of such patients exhibit pragmatic deficits, the relevant studies could not provide an explanation about the formation of the aforementioned subgroups which demonstrated distinct profiles. This could be due to a lack of detailed information about brain lesions and limited evaluation of other cognitive functions. Consequently, it has not yet been established why these differences among RBD patients exist and what is the exact nature of their cognitive profiles [2].

To address the second factor (i.e., the aforementioned pathological mechanisms), we will attempt to disentangle the issue at hand, by briefly discussing the underlying cognitive mechanisms of pragmatics and their possible relations to other cognitive domains and constructs. The first cognitive construct that has been associated with pragmatics is Theory of Mind (ToM), which refers to making inferences about ourselves and others [31,32]. Several studies have highlighted the relationship between pragmatics and ToM [33,34], arguing that ToM is necessary for recognizing the mental and emotional state of others as a critical factor for understanding what they are implying [27]. However, the actual data are limited and usually focused on false belief tasks and pragmatics [35], while leaving out other subcomponents of the relevant cognitive mechanisms. In addition, research indicates that pragmatics and ToM do not have a linear relationship, with the suggested explanatory hypotheses varying; it has been argued that pragmatics serve as a subcomponent of ToM, that pragmatics and ToM refer to distinct, but interrelated or overlapping, cognitive mechanisms [36,37], or even that some components of ToM may not be related to non-literal language at all [24] and some pragmatic components rely on different cognitive strategies besides ToM [38,39].

The second cognitive domain associated with pragmatics, namely executive functions (EFs), refers to mental skills necessary to regulate our behavior and may include—among others—inhibition, mental shifting, working memory, and attention [40]. Stroke patients often demonstrate EF deficits [41,42,43,44] and in some cases these may be associated with pragmatic impairments. For instance, inhibition and cognitive flexibility seem to play an essential role in understanding the secondary meaning of a word or a sentence. This assumption is based on the hypothesis that an inability to inhibit the primary meaning of an expression, e.g., the literal meaning, and difficulty in cognitive flexibility do not allow the person to shift from one meaning (the literal) to another (the non-literal) [27,45,46]. Deficits in working memory and attention have also been assumed to contribute as underlying pathological mechanisms resulting in pragmatic deficits [47]. However, other studies have failed to show strong links between executive functions and pragmatic deficits. For example, Champagne and colleagues [48] found a correlation between pragmatics and inhibition, but not shifting. Bambini and colleagues [49] found that different aspects of communication may rely on different executive mechanisms, such as working memory and to some extent cognitive flexibility, but not inhibition, in healthy populations. Finally, there are studies which failed to find any relation between EF and pragmatic deficits in RBD patients [50]. Given the complex nature of EF and pragmatics and the lack of consensus on findings, further analysis of the relationship between these two mechanisms is deemed necessary.

So far, only two studies have investigated the combination of these three components (ToM, EF, and pragmatics) in patients with right hemisphere stroke, and their results are controversial regarding their relation. The first study showed that ToM is mainly related to pragmatic deficits and executive functions play a role only when combined with ToM deficits [25]. The second study showed that, although both components play a role in pragmatic deficits, this correlation was not enough to explain the communication deficits [28], and the relationship between pragmatic skills and underlying cognitive mechanisms seems to be ambiguous. A possible explanation for this ambiguity is that ToM and executive functions might predict different aspects of pragmatics [24].

Although studies on compromised underlying cognitive mechanisms have provided indications regarding how pragmatic deficits may emerge in RBD patients, there is lack of consensus about the exact nature of the link between pragmatics and cognitive mechanisms, and consequently the corresponding profiles of RBD patients are not easily interpreted [35].

In sum, although there are indications that RBD patients exhibit different profiles of pragmatic deficits, the underlying cognitive functions, which could serve as an explanatory framework, are not yet well understood [29,35]. Moreover, the relationship between right hemisphere damage and pragmatic deficits is generally unclear, as it has not been yet confirmed whether there is any association pattern between specific lesion loci and impaired performance on pragmatic tests [20,35]. While functional imaging studies in neurotypical populations have highlighted the importance of the right hemisphere for pragmatics (e.g., the processing of metaphors), there are no sufficient data from lesion studies [4,7,51].

The present study aims to clarify the relation among pragmatic skill deficits, Theory of Mind, and executive functions and to investigate possible lesion correlates of pragmatic deficits in RBD patients. We hypothesize that different pragmatic subcomponents are associated with different cognitive impairment profiles, which might explain the heterogeneity of pragmatic deficits in patients with right hemisphere stroke. Possible relationships of the above profiles with lesion topology are also investigated.

## 2. Methods

### 2.1. Participants

Twenty-five RBD patients (10 women) (mean age 58) and thirty-seven healthy participants as a control group (19 men) (mean age 52) were recruited for this study. Participants were matched for age (Z = −0.826, *p* = 0.41) and gender (x^2^ = 0.772, *p* = 0.38). All RBD patients suffered from a single stroke lesion and the examination took place at least 6 months post stroke. Structural imaging data (non-digital CT and/or MRI scans) were obtained for 21 patients and lesions were identified and coded by a neuroradiologist blind to the neuropsychological results as described in [52]. Patients’ characteristics are shown in Table 1. All participants were right-handed (handedness established through detailed clinical/personal history) and Greek native speakers with no previous psychiatric or neurological disorder. Patients were tested with the neuropsychological battery described below. Informed consent was obtained from all participants prior to participation. The testing was conducted at Eginition Hospital in Athens, School of Medicine, Greece (research protocol approval ID: 7ΓOΜ46Ψ8Ν2-8ΘΜ). The battery consisted of 4 tests for the evaluation of the executive functions, 2 Theory of Mind tests, 2 tests which evaluate the pragmatic aspect of language, and, in order to rule out any cases of crossed aphasia, 2 tests for the evaluation of naming and comprehension of language. Controls were tested with the tests assessing ToM and pragmatics, in order to derive cutoffs based on expected healthy performance, since normative data for such tests are lacking for the Greek population.

### 2.2. Materials

#### 2.2.1. Executive Functions

The Greek standardized version of the Stroop test was used for the evaluation of inhibition control. The test consists of 2 lists of 120 color words. For the first list, the trial condition, the participant is asked to read the list of words as fast as possible, while for the 2nd list the participant is asked to name the color of the words [53]. The digit span was used to evaluate the short-term and working memory. This test consists of two conditions, forward and backward. In the first condition, the examiner reads a list of numbers and the examinee is asked to recall them with the exact same order. In the 2nd condition, the examiner reads a list of numbers and this time the examinee is asked to repeat them in reverse [54]. For processing speed, the symbol digit modality test (SDMT) was used. In this test, the examinee is given a page with a key consisting of 9 numbers and 9 corresponding symbols. The examinee must then fill in the correct number for the respective symbol, within 90 s [55,56]. For the evaluation of cognitive flexibility, the Greek standardized version of the trail-making test was used. This test consists of 2 conditions. In the first condition, the examinee is asked to make a serial connection of the numbers in ascending order as quickly as possible. In the 2nd condition, which evaluates mental flexibility, the examinee is asked to draw a line through repeated alternations of numbers in ascending order and letters in alphabetic order [57]. A derived index (i.e., time for completion of TMT-B minus time for completion of TMT-A) was calculated and used for subsequent analyses and case-by-case investigation (please see Section 3) following the guidelines by [58].

#### 2.2.2. Pragmatics

To assess indirect speech comprehension, we developed a 20-sentence test, based on the Montreal battery of communication deficits—Protocole Montréal d’ Évaluation de la Communication [59]. The test was designed to be administered to a Greek-speaking population and therefore it was required to create new stories based on the social and cultural peculiarities of native Greek speakers. A total of 20 sentences were constructed, 10 of which contained an indirect request, with a minimum score of 0 and maximum score of 40 (each item’s score ranged from 0 to 2).

To evaluate the comprehension of non-literal language, a test of a total of 26 metaphors was designed, with a minimum score of 0 and maximum score of 52 (each item’s score ranged from 0 to 2). The construction of this test was based on the test of understanding the metaphorical speech of the Montreal battery of communication deficits—Protocole Montréal d’ Évaluation de la Communication [59]. The test was designed to be administered to a Greek-speaking population and therefore it was required to choose and create new metaphors based on the social and cultural peculiarities of native Greek speakers. Examples of the material are shown in Box 1.

Box 1 **Examples of the material in the pragmatics tests.**

**Example for metaphor interpretation:** The mother is an angel.

 

Example for indirect request interpretation

Maria enters the office and sees the window closed. She tells her colleagues: “It’s hot in here!”

(A) Maria is stating the room temperature.

(B) Maria wants someone to open the window.

#### 2.2.3. Language

For assessing lexical retrieval, the short Greek version of the Boston naming test was used [60,61]. Comprehension of Instructions in Greek (CIG) was used to evaluate complex commands [62].

#### 2.2.4. Theory of Mind

For the evaluation of Theory of Mind abilities, one non-verbal and one verbal test were used. For the non-verbal condition, the Frith–Happé animation test was used. The test consists of a total of 12 videos, lasting approximately 45 s, in which two triangles, a small blue one and a large red one, interact with each other. Videos are divided into 3 categories of interaction: (A) random movement, (B) goal-directed, and (C) ToM. In the first condition, there is no interaction between the two geometric shapes. In the second condition, the interaction between the two shapes is purely kinetic–physical, like one shape copying the other, and in the third condition the interaction is mental–emotional, e.g., one triangle is trying to mock the other one. The participant is asked to describe what is happening in the videos, categorize the type of interaction (cognitive facet of ToM) of the test with the minimum score being 0 and maximum score being 10, and answer multiple-answer questions (affective facet of ToM) with the minimum score being 0 and maximum score being 8 [63,64]. For the verbal condition, the Greek brief version of the faux pas test, adapted by Patrikelis and Angelakis (translation available at the Autism Research Centre website: autismresearchcentre.com), was used to assess affective and cognitive ToM and cognitive empathy. The test contains 5 faux pas stories and 5 control stories. The score of each story has a minimum value of 0 and maximum value of 30, the cognitive subcomponent has a minimum score of 0 and maximum score of 10, the affective component has a minimum score of 0 and maximum score of 5, and the cognitive empathy component has a minimum score of 0 and maximum score of 5. The participant is first exposed to these stories, and then they must recognize if there is any socially inappropriate content, who committed this inappropriate act (recognition of faux pas), what were the intentions of the protagonist (cognitive ToM), and what were the feelings of the protagonists (affective ToM and cognitive empathy) [65].

## 3. Data Analyses

Preliminary investigation with the Shapiro–Wilk criterion revealed violations of normality, and thus we proceeded with non-parametric analyses. Therefore, we compared the two groups using the Mann–Whitney U test. Then, we conducted a case-by-case investigation- see (Table 2). First, we transformed patients’ raw values into z-scores, based on published normative data for the executive [53,56,57,58] and language tests [60,61,62]. Since there are no available Greek normative data for the pragmatics and ToM tests, we derived percentiles based on our control group’s data for these measures. Based on the patients’ standardized values (for the executive and language tests) and the aforementioned percentiles (for the ToM and pragmatics tests), we designated each patient as impaired or non-impaired in the following cognitive components: affective non-verbal ToM, cognitive non-verbal ToM, affective verbal ToM, cognitive verbal ToM, verbal short-term memory, verbal working memory, processing speed, inhibition, cognitive flexibility, pragmatics—metaphors, pragmatics—indirect requests, naming, and auditory comprehension. The cutoffs were the 5th percentile or a z-score equal to or below −1.5 standard deviations, depending on the test. We then classified patients into subgroups on the basis of their impairment into three main cognitive domains, namely executive functions, pragmatics, and ToM. Subsequently, two separate hierarchical regression models were conducted for the RBD group, using factors based on zero-order correlations (including behavioral and demographic variables) and then partial correlation (between behavioral measures, controlling for years of formal schooling, a demographic variable which was significantly correlated with several behavioral measures—see Table 3), in order to investigate whether performance on pragmatic tests could be predicted by specific variables, indicated by the preliminary correlation analyses. The first model included education and cognitive ToM as independent variables and indirect requests as the dependent variable. The second model included education, Stroop test, digit span forward, and affective ToM as independent variables and metaphors as the dependent variable.

Finally, a cluster analysis was conducted. Since behavioral data could not be normalized or transformed into z-scores, cluster analysis with Pearson correlation was chosen. This method is less influenced by magnitudes, thus allowing variables with different magnitudes to be included. The cluster analysis was performed on the behavioral data with the following clustering conditions: clustering the objects, utilizing the group average cluster method, employing Pearson correlation as the distance type, and determining the clustroid based on the sum of distances. Group comparisons were performed using IBM SPSS 22.0 and cluster analysis was performed with OriginPro, Version 2023b (OriginLab Corporation, Northampton, MA, USA).

## 4. Results

**Figure 1:**
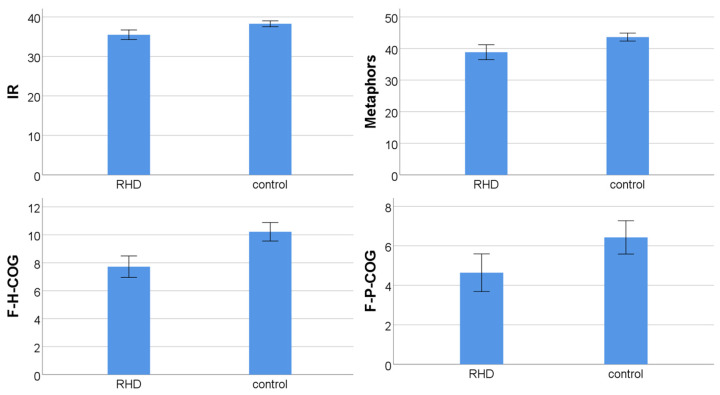
Bar charts showing significant difference between the two groups regarding their performance on the four psychometric tools (error bars: 95% CI); IR: indirect requests; F-H-COG: Frith–Happé categorization (cognitive condition of ToM); F-P-COG: faux pas (cognitive condition of ToM).

Mann–Whitney U tests revealed that the RBD group performed worse on both pragmatic tests (Z = −3.878, *p* = 0.0001 and Z = −3.279, *p* = 0.001 for indirect requests and metaphors, respectively) and two ToM scores (Z = −4.391, *p* = 0.00001 for Frith–Happé cognitive condition of ToM and Z = −2.616, *p* = 0.009 for the cognitive condition of faux pas test). The differences described above can be seen in Figure 1. No significant differences were found for the affective and empathy components of the faux pas tests (Z = −1.701, *p* = 0.089 and Z = −0.754, *p* = 0.451, respectively), while a marginal difference was found for the affective condition of the Frith–Happé test (Z = −2.010, *p* = 0.044). Significant differences were observed for all executive measures (for Stroop Z = −3.757, *p* = 0.0001; for DS forward Z = −3.063, *p* = 0.002; for DS backward Z = −2.787, *p* = 0.005; for SDMT Z = −4.724, *p* = 0.000002; for TMT Z = −3.912, *p* = 0.00009). The subsequent case-by-case investigation showed that besides the fact the RBD patients as a group seem to be impaired in these two pragmatic subcomponents, not all patients exhibit pragmatic skill deficits, revealing distinct profiles, with the majority of patients having impairment in the metaphorical interpretation task, other patients in the indirect request comprehension task, and a third profile of patients having deficits in both pragmatic tasks, showing the heterogeneity of the RBD patients regarding their pragmatic skills impairment and the deficits in ToM and executive functions.

The outcome of the case-by-case investigation is shown in Table 2. There were 20 patients with EF deficits, 15 patients with pragmatic deficits, and 16 patients demonstrating ToM deficits. Ten out of twenty-five patients demonstrate deficits in all three domains, four patients demonstrate deficits in pragmatics and executive functions, and only one patient exhibits deficits in ToM and pragmatics. Three patients demonstrate deficits in EF and ToM without any pragmatic impairment. Besides this, two patients do not demonstrate any deficit at all. Last, but not least, all the patients who exhibit some type of pragmatic deficit also exhibit an impairment in at least one of the other two cognitive mechanisms, i.e., ToM and EF, besides one patient who exhibits a borderline score in inhibition and no other impairment is detected. Two experienced neuropsychologists confirmed that there was no prominent language disturbance in our sample (i.e., crossed aphasia), after clinical examination of the patients and careful evaluation of their neuropsychological profiles. In some cases, there were low scores on naming and auditory comprehension measures, which were attributed to executive and/or immediate memory impairment [66,67,68,69].

The results of the first regression analysis indicated that increased performance in the Frith–Happé cognitive condition of ToM (β = 0.509, t = 3.339, *p* = 0.00146) can significantly predict better performance in indirect requests, when education is held constant (see Table 4).

In the second model using metaphors as a dependent variable, results indicated that increased years of education (β = 0.572, t = 2.678, *p* = 0.010) can only predict better performance in metaphors when performances in the Frith–Happé affective condition of ToM, Stroop test, and digit span forward are held constant. It should, however, be noted that when the Frith–Happé affective condition of ToM was entered at the second step of the hierarchical regression, significant results were observed for both the corresponding beta coefficient and the R^2^ change (see Table 5).

**Figure 2:**
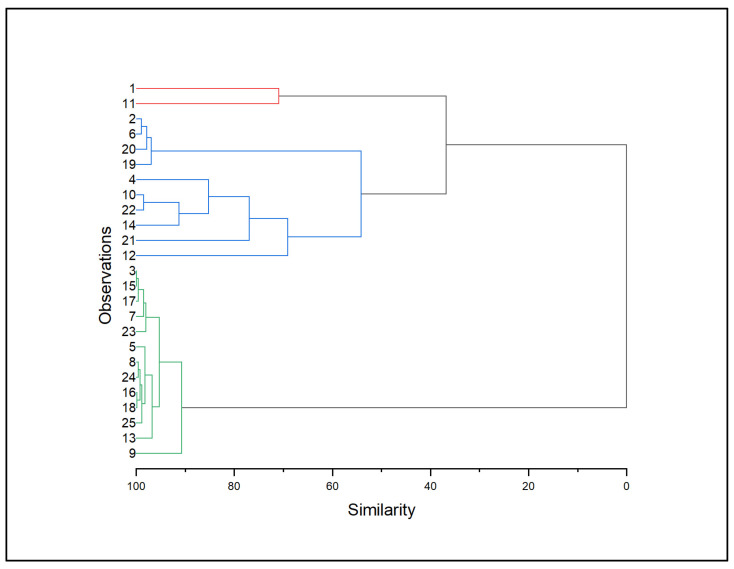
The cluster analysis of the behavioral data presented in a dendrogram. As explained in the text, there are two main clusters. One with ten RBD patients, who mainly demonstrate ToM deficits (blue), and one with thirteen RBD patients, most of whom exhibit impaired performance in all three cognitive domains (green).

For the cluster analysis, the results are shown in Figure 2. As is shown, behavioral data form two main clusters based on their correlations. Furthermore, subjects 1 and 11 should be studied separately due to their low similarity with the other two clusters. The second cluster consists of ten patients (2, 6, 20, 19, 4, 10, 22, 14, 21, 12). In this cluster, the patients exhibit mainly ToM deficits, specifically in the affective facet of the non-verbal test, besides patient 10 who exhibits deficits in three subcomponents from the two ToM tests, with some also presenting impairment in executive functions, specifically in the processing speed. The third cluster consists of 13 patients, 3, 15, 17, 7, 23, 5, 8, 24, 16, 18, 25, 13, 9. The patients of this cluster exhibit a generalized cognitive dysfunction. In this group, 9 out of 13 patients exhibit deficits in all three domains, including deficits in the pragmatic subcomponents, besides patients 7 and 8 and 15 who do not have an impairment in ToM (see also Table 2).

## 5. Discussion

The present study aimed to investigate the clinical profiles of patients with right hemisphere damage (RBD), regarding the nature of their communication deficits. Participants were evaluated in two pragmatic tests (e.g., metaphors and indirect speech), in executive functions, ToM, and other language tests. Additionally, lesion loci were identified and coded for each participant.

Our results showed that RBD patients performed lower as a group in pragmatic tests compared to the control group. Therefore, the results confirm previous studies, which have shown that RBD patients exhibit communicational deficits [22,48]. A further case-by-case investigation revealed that not all patients demonstrate pragmatic impairments, a finding that verifies the heterogeneity of the patients in terms of communication deficits, a result that has been previously suggested [20,30]. However, 10 of 25 patients demonstrated a deficit in all three cognitive domains (ToM, EF, and pragmatics). This result shows that besides the heterogeneity of the RBD patients, many exhibit a distinct cognitive profile regarding their difficulties in pragmatic aspects of language, with a co-occurrence of both ToM and executive functions. On the other hand, only four patients exhibit pragmatic deficits combined with only one of the two underlying cognitive mechanisms, and no patient exhibits pragmatic deficits without either of the two other cognitive functions mentioned above, with the exception of one patient who exhibits a borderline deficit score in inhibition, without any other cognitive function being impaired. The fact that there was no patient with isolated pragmatic impairment indicates that at least one other component (either executive function or ToM) needs to be compromised in order for a deficit in a pragmatic component to emerge [25]. Moreover, it seems that RBD patients show different patterns of cognitive performance regarding their pragmatic skills. Specifically, 15 of the 25 patients exhibited pragmatic deficits. Twelve out of twenty-five RBD patients revealed low performance in the metaphor comprehension task, and six out of twenty-five in the comprehension of indirect speech task. Among these patients, only three of them presented low performance in both pragmatic paradigms. The type of pragmatic difficulties seems to vary among the patients.

The existence of RBD subgroups on the basis of communication abilities has been discussed in the literature [30], finding different groups based on their deficits in linguistics tasks, such as comprehension of irony, extralinguistic tasks namely comprehension of language via non-verbal communication, and paralinguistic tasks, i.e., prosody [30]. Moreover, Champagne-Lavau and Joanette [25] found different patterns of communication deficits, explaining that the pragmatic deficits were mainly associated with the performance in ToM tasks. Despite the observed heterogeneity, a general trend seems to emerge, with a quite large subgroup of our patients with pragmatic difficulties exhibiting deficits in both executive functions and ToM. That is why we attempted to investigate possible relationships between impairment in the two latter domains and pragmatic deficits.

Several studies, in various clinical populations and healthy participants, have shown different results regarding the relationship among pragmatics, executive functions, and ToM, with different research groups reporting various results regarding the relationship among these three variables [49,70,71]. In our study with RBD patients, regression analyses revealed distinct cognitive mechanisms that underlie the performance with metaphors and indirect requests, with a common variable being the education level. Notably, level of education seems to be a common predictor for both pragmatic subcomponents. This result is confirmed by previous research on pragmatics, in healthy individuals indicating that the level of education can partially influence the performance of processing of pragmatic cues [72]. Regarding cognitive mechanisms, metaphors seem to be predicted mainly by executive functions and affective ToM. This underscores that the process of metaphorical speech is supported by both cognitive functions, with executive functions—in particular, inhibition—having a pivotal role. Regarding inhibition, it could be hypothesized that it is an important factor for metaphor interpretation since the listener might need to inhibit the primary, literal meaning of the word/sentence and process the language cue based on a secondary, non-literal meaning [28]. On the other hand, regarding the affective ToM, metaphors have been shown to be associated with emotional impact, surpassing literal phrases, and even creating affective response in the brain [73]. This phenomenon might arise from the inherent emotional weight that certain words produce when employed metaphorically. Furthermore, metaphors have been associated with participants exhibiting higher scores in affective ToM assessments subsequent to exposure to metaphorical sentences. Reading metaphors can cause a shift towards focusing on interpersonal social information while performing an affective ToM test [74,75]. Thus, we could hypothesize that it is essential for the listener to be able to attribute the emotional state of the interlocutor in order to understand the way a phrase has been expressed. A deficit in the effective facet of ToM might reduce this ability.

On the other hand, indirect requests seem to be predicted by cognitive ToM and no relation was found with any executive functions, showing that the ability to attribute the intention and the perspective of the speaker is needed in order to understand an indirect request. Therefore, based on our results, the metarepresentation of the phrase in a context is the main ability needed to process an indirect speech act [25]. Indirect requests and metaphors have different linguistic structures, thus having different underlying cognitive mechanisms supporting the two pragmatic subcomponents [76,77,78]. This dichotomy is an indication that different pragmatic impairments are derived from different patterns of cognitive deficits. Since pragmatics has a very heterogenous language structure, this result could explain at least partially the research so far on pragmatic skill deficits and why the cognitive mechanisms are ambiguous.

Regarding the cluster analysis, three distinct subgroups (i.e., clusters) of patients are revealed. The first cluster, consisting of two patients, exhibits low similarity to the rest of the group and no specific pattern can emerge. The second cluster consists of patients who exhibit mainly ToM deficits with some also presenting impairment in executive functions. However, the deficits in the executive functions are mainly seen in the processing speed, which is distantly related to pragmatic skills. As for ToM, almost all patients in this cluster exhibit deficits in just one subcomponent of the cognitive function, specifically in the affective component of the non-verbal ToM test. Therefore, it could be hypothesized that a specific deficit in one ToM facet cannot explain a generalized pragmatic impairment. On the other hand, the 3rd cluster consists of patients with more diffuse deficits in the cognitive functions assessed in this study. In particular, nine out of thirteen patients exhibit deficits in all three domains, besides one patient who does not exhibit pragmatic skills deficit at all and three patients who exhibit deficits in metaphors and executive function, without having impairment in ToM. Overall, the patients of the 3rd cluster show a general tendency to exhibit deficits in multiple domains, reflected by impaired performance on several tasks linked to various facets of ToM, executive functions, and pragmatics.

Adding to that, an interesting outcome that derives from this study is that, as mentioned above, all patients who exhibit some kind of pragmatic deficit also exhibit low performance in at least one of the other two cognitive functions (i.e., ToM and EF). Although the relationships among ToM, EF, and pragmatics remain ambiguous, [35], we can conclude that the integrity of these two cognitive mechanisms (i.e., EF and ToM) seems to be crucial for pragmatic processes. In this sense, pragmatics cannot be viewed as a language subcomponent of ToM [36,37], since executive functions are involved independently of ToM [46,49]. The observed pragmatic deficits in our study seem to stem from two different types of impairment: the first affecting both affective and cognitive facets of ToM, which are necessary for in-context comprehension, and the second affecting inhibition, a restraining executive mechanism essential for inhibiting irrelevant responses. The specifics about the unique contribution of these two types of impairment remain to be elucidated but it may depend on the pragmatic subcomponent examined.

A remaining question concerns the relationship between lesion sites and pragmatic deficits. Although some data are available on the relationship between brain regions and pragmatics from studies with healthy participants [79,80], studies involving brain-damaged patients are scarce. The most frequent lesion locus in the group of patients who exhibit deficits in all three cognitive domains is the inferior parietal lobule (seven out of nine patients), followed by frontal areas such as middle frontal gyrus (six out of nine patients), pars triangularis and pars opercularis (five out of nine patients), insula (six out of nine patients), and precentral gyrus (seven out of nine patients). Based on the data derived from the cluster analysis, the third cluster of patients seems to exhibit systematically more lesions in the aforementioned areas compared to the patients of the second cluster, besides the IPL which is equally distributed in clusters 2 and 3. The inferior parietal lobule seems to be a crucial area for ToM, while it has also been linked to executive functions. ToM has been shown to be associated with a network involving the dorsomedial prefrontal cortex, (dmPFC), precuneus, temporoparietal junction (TPJ), orbitofrontal cortex (OFC), and parts of the limbic system [81,82]. The inferior parietal lobule (IPL) appears to be involved in attributing beliefs to someone else; in particular, the temporoparietal junction is argued to be a crucial area for predicting reason, beliefs, desires, and intention of other people (Saxe & Kanwisher, 2003). The inferior parietal lobule (IPL) also seems to be involved in the executive control of behavior, particularly inhibition [83]. Concerning the frontal lobe lesions of these patients, the literature suggests that the middle frontal gyrus (MFG), the insula, and the posterior part of the inferior frontal gyrus seem to have a role in a plethora of executive components [84,85]. Another area of interest is the precentral gyrus; while it does not seem to be involved directly in attributing mental states to others, it seems to be essential for distinguishing one’s perspective from those of others [86], which might be important for the in-context comprehension of a phrase. The aforementioned areas seem to be connected via white matter tracts. In particular, the IPL appears to be connected with the MFG, insula, and inferior frontal gyrus (IFG) via the superior longitudinal fasciculus (SLF) and the arcuate fasciculus (AF) [87] and the middle frontal gyrus with the inferior part of the precentral gyrus via the AF [87]. Besides this, fMRI studies investigating the neural basis of pragmatics have highlighted specific right hemisphere brain areas of interest. Regarding metaphor processing, it has been suggested that right-lateralized regions, including the insula, premotor cortex, pars triangularis, pars opercularis, precuneus, and prefrontal and temporal cortices are involved [4,88]. As for processing of idioms, it has been argued that the inferior frontal and middle temporal gyri of the right hemisphere are involved [89].

Moreover, the lesions of cluster 3 patients affect brain regions which are known to be involved in the default mode network, the dorsal attention network, the salience network, and the language network. These networks have been associated with social cognition and pragmatic processing in both pathological and healthy populations [90,91,92,93]. Although the investigation of such networks is beyond the scope of this study, it could be speculated that a disruption of these networks could aid in explaining the constellation of cognitive deficits of RBD patients who demonstrate impaired pragmatic processing.

In sum, we put forward a working hypothesis suggesting that a widely distributed network consisting of right-lateralized posterior and anterior regions might be involved in the interpretation of the pragmatic aspect of language; the main component of this network seems to be the IPL while the MFG, insula, and posterior frontal cortices (including the pars opercularis and the pars triangularis) may serve as subcomponents. Lesions affecting the aforementioned brain regions could result in pragmatic deficits due to the involvement of the above-mentioned areas in cognitive mechanisms essential for understanding the intentions and the perspective of the speaker, as well as executive aspects, such as the ability to inhibit the primary, literal response or mental switching to the appropriate non-literal meaning.

## 6. Conclusions

The present study aimed to investigate the relationship between pragmatic impairment and right hemisphere damage and the possible role of deficits in executive functions and Theory of Mind. The findings of the study provided insights into the relationship between RBD and pragmatic deficits and the possible contribution of impaired cognitive domains. The detailed case-by-case investigation revealed the heterogeneity of RBD patients regarding their pragmatic deficits. Moreover, we observed a frequent co-morbidity of deficits in pragmatics, ToM, and executive functions, and the IPL was shown to be a common lesion site in such patients. Our regression analyses also revealed that different pragmatic subcomponents may be predicted by different underlying cognitive mechanisms, a finding that could offer an explanation of the heterogeneity of RBD patients with respect to their pragmatic impairment. Lastly, our cluster analysis generally confirmed our preliminary case-by-case investigation, indicating that a pragmatic impairment will most probably result after both EF and ToM components have been compromised in RBD patients. Overall, we postulate that there is an involvement of ToM and executive functions in pragmatics in the sense that a pragmatic impairment may stem from deficits in these two cognitive domains due to right hemisphere lesions, making it a social–cognitive impairment rather than a typical language deficit, affecting a widely distributed network in which IPL seems to play a central role. Although this hypothesis is intriguing, its possible implications should be taken with a grain of salt, in the sense that our sample was relatively small and lesion localization in our study was based on non-computerized methods. Moreover, we assessed pragmatics with only two tests, even though we acknowledge that it is probably a multi-component construct rather than a unitary function. In any case, our findings provide a useful insight towards understanding pragmatic impairment, its association with executive and ToM deficits, along with their lesional substrate. Future research with larger samples, comprehensive neuropsychological batteries covering a wide range of aspects of pragmatics, ToM, and EF, as well as more sophisticated imaging techniques could shed light on this complex issue about the emergence of supralinguistic communication difficulties following right hemisphere lesions.

## Figures and Tables

**Table 1:** Demographic characteristics and lesion data for the RBD patients.

Patient	Gender	Age	Education	Lesion Loci
1	M	48	12	PrG
2	M	50	12	IC
3	M	76	16	Missing
4	M	74	12	Missing
5	F	61	6	IPL, STG/MTG
6	F	20	15	IC, Th
7	M	51	12	IC, Ins, Pt-Po, IPL, STG/MTG
8	F	58	14	SMA, PrG, MFG
9	M	64	17	PrG, Ins, Pt-Po, MFG
10	F	64	14	IPL
11	M	65	9	IC, EC, GP, Ptm, CN, Th, PrG, Ins, Pt-Po, MFG, IPL, STG/MFG
12	M	59	24	IC, EC, Ptm, Th, SMA, Ins, Pt-Po, MFG, IPL
13	M	81	9	SMA, PrG, Ins, IPL
14	F	55	12	IC, EC, Th
15	F	55	16	Missing
16	F	53	14	PrG, Pt-Po, MFG, IPL
17	M	58	14	SMA, PrG, Ins, Pt-Po, MFG, IPL
18	M	47	9	EC, SMA, Ins, IPL
19	M	45	18	Missing
20	F	53	12	GP, Ptm, Th, SMA, PrG, IPL
21	M	70	12	IC, EC, GP, Ptm, CN, Th, IPL
22	M	62	16	GP), Ptm, CN, SMA, PrG, Pt-Po, MFG, IPL, STG/MFG
23	F	69	16	IC, CN, Th
24	F	55	6	PrG, MFG, STG/MTG
25	M	67	12	IC, EC, GP, Ptm, CN, Th, SMA, PrG, Ins, Pt-Po, MFG, IPL

CN: Caudate Nucleus; EC: External Capsule; GP: Globus Pallidus; IC: Internal Capsule; IPL: Inferior Parietal Lobule; Ins: Insula; MFG: Middle Frontal Gyrus; PrG: Precentral Gyrus; Pt-Po: Pars Triangularis–Pars Opercularis; Ptm: Putamen; SMA: Supplementary Motor Area; STG/MTG: Superior Temporal Gyrus/Middle Temporal Gyrus/Superior Temporal Sulcus; Th: Thalamus.

**Table 2:** Individual patient performance in the three cognitive domains.

Patient	Cluster	Pragmatics ^1^		Verbal ToM ^1^		Non-Verbal ToM ^1^		Executive Functions ^3^				
		Met ^2^	IR ^2^	Cog ^2^	Aff ^2^	Cog ^2^	Aff ^2^	Inhibition ^2^	STM ^4^	WM ^4^	CogFlx ^5^	PrSp ^6^
1	1	0.8	1	0.7	0.8	0.8	0.8	0.4 *	5	4	69	25 *
11		0.6 *	0.8 *	0.3 *	0.6	0.3 *	0.6	0.2 *	3 *	3 *	300 *	29
2	2	0.8	0.9	0.2 *	0.6	0.8	1	1	6	5	50	46
6		0.9	0.9	0.9	1	0.6	1	1	6	6	46	55
20		0.8	0.9	0.7	0.8	0.6	1	0.9	7	4	54	43
19		0.9	1	0.8	0.8	0.9	0.6	0.9	7	5	77	35 *
4		0.7 *	1	0.5	0.6	0.8	0.5 *	0.8	7	6	155	27
10		0.8	0.9	0.2 *	0.8	0.3 *	0.5 *	0.7	5	3	157 *	26 *
22		0.8	0.9	0.6	1	0.7	0.4 *	0.9	5	5	116 *	23 *
14		0.8	0.8 *	0.5	0.6	0.7	0.8	0.7	5	3	102	42
21		8	0.9	0.4	1	0.6	0.5 *	0.3 *	5	3	111	30
12		0.8	0.9	0.6	1	0.9	0.5 *	0.7	5	4	70	0 *
3	3	0.7 *	0.9	0.7	1	0.7	0.9	0.6	4 *	3 *	231 *	38
15		0.8	1	0.4	0.6	0.7	0.6	0.7	5 *	4 *	300 *	42
17		0.8	0.7 *	0.4	0.8	0.4 *	0.4 *	0.8	4 *	2 *	300 *	34
7		0.7 *	0.9	0.6	1	0.6	0.6	0.5	6	4	175 *	27
23		0.8	0.8 *	0.6	0.6	0.6	0.5 *	0.8	4 *	3	300 *	26 *
5		0.7 *	0.8 *	0.1 *	0.2 *	0.8	0.4 *	0.6	5	3	300 *	7 *
8		0.6 *	0.9	0.7	1	0.7	0.6	0.4	4	3 *	300 *	11 *
24		0.4 *	0.9	0.6	0.8	0.6	0.1 *	0.4 *	4 *	4	300 *	13 *
16		0.7 *	1	0.2 *	0.2 *	0.5 *	0.8	0.3 *	5 *	4	300 *	10 *
18		0.7 *	0.9	0.1 *	0.4 *	0.6	0.8	0.4 *	4	3	300 *	8 *
25		0.5 *	0.8 *	0.4	0.6	0.6	0.5 *	0.4 *	4 *	4	300 *	24
13		0.6 *	1	0.2 *	0.2 *	0.7	0.5 *	0.3 *	6	3 *	300 *	38
9		0.7 *	0.9	0.2 *	0.6	0.7	0.3 *	0.3 *	5 *	4 *	134 *	29

Met: Metaphors; IR: indirect requests; Verbal ToM: performance on the faux pas test; Non-Verbal ToM: performance on the Frith–Happé animation test; Cog: cognitive dimension of ToM; Aff: affective dimension of ToM; STM: short-term memory assessed by forward digit span; WM: working memory assessed by backward digit span; CogFlx: cognitive flexibility assessed by the derived trail-making test index; PrSp: processing speed assessed by symbol digit modality test performance. Asterisks denote impaired performance. ^1^ Impaired performance judged on the basis of percentiles derived from the control group. ^2^ The numbers represent accuracy, i.e., number of correct responses relative to maximum possible performance. ^3^ Impaired performance judged on the basis of previously published normative data. ^4^ Numbers represent span (forward in the case of STM and backward in the case of WM). ^5^ Numbers represent the derived trail-making test index (TMT-B minus TMT-A). ^6^ Numbers represent the correct items within the time limit of 90 s. For details about the clusters, please see Section 4 (cluster analysis).

**Table 3:** Partial correlations between patients’ scores in the three cognitive domains, including education as a nuisance variable.

	IR ^1^	Met ^2^	Non-Verbal ToM Cog ^3^	Non-Verbal ToM Aff ^4^	Inhibition	STM ^5^	WM ^6^	Processing Speed	Verbal ToM Cog ^7^	Verbal ToM Aff ^8^	Cog Flex ^9^
IR ^1^	1.000										
Met ^2^	0.051	1.000									
Non-Verbal ToM Cog ^3^	0.443 *	0.162	1.000			.					
Non-Verbal ToM Aff ^4^	0.227	0.532 *	−0.027	1.000							
Inhibition	−0.128	0.629 **	0.095	0.350	1.000						
STM ^5^	0.359	0.489 *	0.304	0.366	0.545 **	1.000					
WM ^6^	0.349	0.246	0.316	0.261	0.479 *	0.712 ***	1.000				
Processing Speed	−0.019	0.390	−0.199	0.503 *	0.478 *	0.412	0.175	1.000			
Verbal ToM Cog ^7^	−0.007	0.035	0.133	0.240	0.265	0.140	0.294	0.256	1.000		
Verbal ToM Aff ^8^	−0.126	−0.070	−0.080	0.073	0.239	0.107	0.240	0.160	0.753 ***	1.000	
Cog Flex ^9^	−0.028	−0.327	−0.047	−0.320	−0.244	−0.397	−0.455 *	−0.282	−0.267	−0.381	1.000

* *p* < 0.05, ** *p* < 0.01, *** *p* < 0.001, ^1^ IR: indirect requests, ^2^ Met: metaphors, ^3^ Non-Verbal ToM Cog: performance on the Frith–Happé animation test—cognitive dimension, ^4^ Non-Verbal ToM Aff: performance on the Frith–Happé animation test—affective dimension, ^5^ STM: short-term memory assessed by forward digit span, ^6^ WM: working memory assessed by backward digit span, ^7^ Verbal ToM Cog: performance on the faux pas test—cognitive dimension, ^8^ Verbal ToM Cog: performance on the faux pas test—affective dimension, ^9^ Cog Flex: cognitive flexibility.

**Table 4:** Results of the first regression model.

Models		B	SE B	β	*p*
1.	Constant	32.506	1.768		<0.001
	Education	0.320	0.120	0.326	0.010
2.	Constant	29.913	1.810		<0.001
	Education	0.176	0.119	0.179	0.145
	Frith–Happé cognitive condition of ToM	0.509	0.152	0.405	<0.001

R^2^ = 0.107, *p* = 0.010 for Step 1; ΔR^2^ = 0.142, *p* < 0.001 for Step 2.

**Table 5:** Results of the second regression model.

Models		B	SE B	β	*p*
1.	Constant	28.974	2.986		<0.001
	Education	0.878	0.202	0.493	<0.001
2.	Constant	25.025	3.287		<0.001
	Education	0.786	0.197	0.442	<0.001
	Frith–Happé affective condition of ToM	1.007	0.410	0.272	0.017
3.	Constant	24.747	3.212		<0.001
	Education	0.572	0.214	0.321	0.010
	Frith–Happé affective condition of ToM	0.695	0.431	0.188	0.112
	Stroop	0.037	0.022	0.215	0.094
	Digit span forward	0.130	0.133	0.123	0.333

R^2^ = 0.243, *p* < 0.001 for Step 1; ΔR^2^ = 0.071, *p* = 0.017 for Step 2; ΔR^2^ = 0.059, *p* ns for Step 3.

## Data Availability

Data are available upon reasonable request to the corresponding author.
